# Guillain-Barre Syndrome, a Leptomeningeal Metastasis Mimic, in a Patient With Metastatic Breast Carcinoma

**DOI:** 10.7759/cureus.37079

**Published:** 2023-04-03

**Authors:** Natalie Torrente, Kimberly Boldig, Anthony Stack, Bharatsinh Gharia

**Affiliations:** 1 Internal Medicine, University of Florida College of Medicine – Jacksonville, Jacksonville, USA; 2 Hematology/Oncology, University of Florida College of Medicine – Jacksonville, Jacksonville, USA; 3 Hematology/Medical Oncology, University of Florida College of Medicine – Jacksonville, Jacksonville, USA

**Keywords:** acute inflammatory demyelinating polyneuropathy (aidp), leptomeningeal metastasis, breast cancer metastasis, breast cancer, guillan-barre syndrome

## Abstract

Leptomeningeal metastasis (LM) is an outcome associated with a terminal prognosis for a patient with metastatic cancer. Symptoms associated with this type of cancer progression can be subtle and nonspecific. Evaluation of LM occurs with a lumbar puncture (LP) and magnetic resonance imaging (MRI). Guillain-Barre Syndrome (GBS) can present with a similar presentation of neurological symptoms to LM. Additionally, both disease states may present with similar MRI findings. The LP can be an important diagnostic evaluation to differentiate LM and GBS. However, an LP may be unremarkable in both disease states. Therefore, a comprehensive assessment of the patient based on clinical history, physical examination, laboratory, and radiologic evaluation is essential for prompt diagnosis and treatment. We present a patient with metastatic breast cancer that presented with generalized weakness. Thorough evaluation allowed the diagnosis and treatment of GBS.

## Introduction

Leptomeningeal metastasis (LM) is the infiltration of the leptomeninges by metastatic carcinoma. It is an uncommon but devastating complication of malignancies such as breast, lung, gastrointestinal, melanoma, primary central nervous system (CNS) cancers, lymphoma, and leukemia [1,2]. It is considered a terminal outcome of metastatic cancer with a median survival of 4.2 months in patients with breast cancer [2]. The incidence of leptomeningeal metastasis varies by primary tumor type, occurring in approximately 5-15% of patients [3]. LM can manifest with a variety of signs and symptoms that involve the central nervous system. In the initial stages of the disease, findings could be subtle and nonspecific, which often leads to a delay in diagnosis. The most common symptoms of LM include headache, nausea, vomiting, neurocognitive deficits, gait changes, hearing loss, visual disturbances, seizures, and dizziness [4]. Treatment options consist of intrathecal chemotherapy and radiation therapy [2].

Other neurologic conditions may present similarly in a breast cancer patient, creating a diagnostic dilemma. Guillain-Barre Syndrome (GBS) is the most common cause of acute flaccid paralysis worldwide, and it initially presents with progressive motor weakness [5]. In population-based studies from North America and Europe, the incidence was noted to range from 0.81 to 1.91 cases per 100,000 person-years (median 1.11) [5]. Two-thirds of patients with GBS report symptoms of a respiratory or gastrointestinal tract infection before the onset of GBS [5]. Campylobacter jejuni is the most common pathogen associated with pathogenesis and is responsible for at least one-third of infections [5].

Imaging findings can also present similarly between the two diagnoses. Both may present with spinal nerve root enhancement on MRI [5,6]. Therefore, it is important to have a high index of suspicion based on clinical presentation and patient history for prompt diagnosis and treatment. We present a case of a patient with metastatic breast cancer whose clinical presentation and diagnostic workup created difficulty in differentiating LM and GBS.

## Case presentation

A 63-year-old female with a past medical history of hypertension, hyperlipidemia, and type 2 diabetes was initially diagnosed with estrogen receptor (ER) positive, progesterone receptor (PR) negative, human epidermal growth factor receptor 2 (HER-2) negative, grade 3, T2N0M0 breast cancer. She was treated with lumpectomy and chemotherapy. Chemotherapy consisted of doxorubicin, cyclophosphamide, and then paclitaxel. She developed toxicity from chemotherapy and was found to have cirrhosis with portal hypertension. Consequently, she was unable to complete chemotherapy. She was also not on adjuvant anti-endocrine therapy due to a history of venous thromboembolism (VTE) and osteoporosis. Two years after the original diagnosis, she presented with recurrent disease and metastasis. Her chemotherapy regimen was changed to fulvestrant and palbociclib. After five months, she developed cytopenia, and palbociclib was discontinued. A positron emission tomography (PET) scan was completed and showed no evidence of new metastatic lesions and resolution of prior metabolically active lesions.

Eighteen days later, she presented to the ER with generalized weakness. She reported two weeks of severe diarrheal illness, nausea, and vomiting, which had resolved upon presentation to the ER. A physical exam showed bilateral muscle weakness and hyporeflexia in the upper and lower extremities. The weakness pattern was symmetric and most prominent distally, in the ankles and hands. Differentials included Guillain-Barré syndrome (GBS) versus leptomeningeal metastatic disease.

A lumbar puncture was performed. Cerebrospinal fluid (CSF) results are listed in Table 1.

**Table 1:** Cerebrospinal fluid via lumbar puncture

		Reference Range
Fluid	Clear	
Red Blood Cell	2	<=0 /UL
White Blood Cell	2	0-10 /UL
Total Protein	39	15-45 MG/DL
Glucose	94	60-80 MG/DL

**Figure 1:**
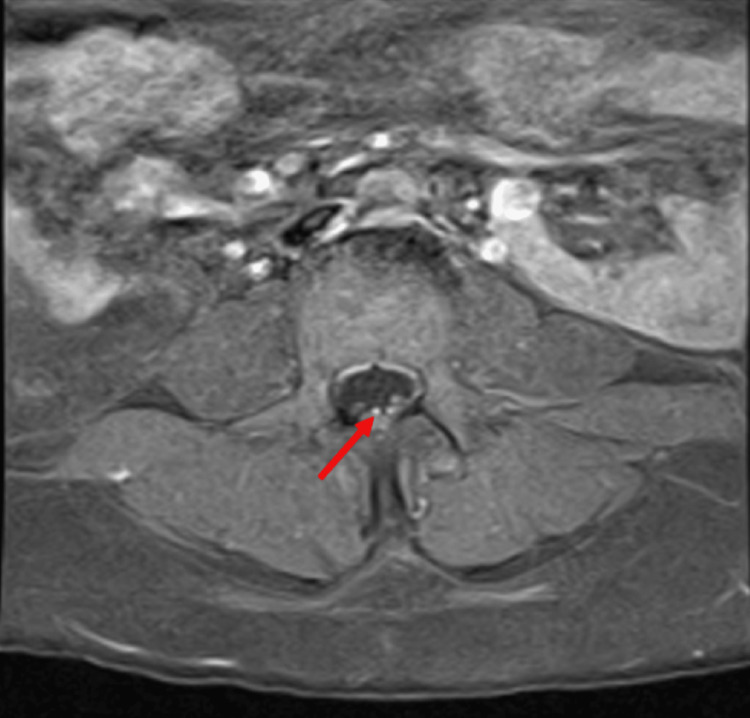
Axial T1 fat-saturated post-contrast image of the lumbar spine demonstrates an enhancement of the cauda equine nerve roots (arrow)

MRI lumbar spine showed abnormal enhancement involving multiple cauda equina nerve roots (Figure 1).

Antibody testing for autoimmune and paraneoplastic causes was negative. However, the clinical history and physical exam indicated a leading differential diagnosis of GBS or acute inflammatory demyelinating polyradiculoneuropathy (AIDP). The patient completed IVIG 400 mg/kg daily for five days. She had gradual improvement in symptoms with intravenous immunoglobulin (IVIG) and continued to improve with inpatient rehab. At follow-up with oncology, a repeat MRI lumbar spine showed a less conspicuous appearance of the cauda equina nerve root as previously described, with no evidence of new or progressive metastatic disease in the lumbar spine. MRI cervical and thoracic spine showed no evidence of metastatic disease.

## Discussion

The pathogenesis of LM was initially described in the 19th century, but recent studies have shown that cancer cells within the CSF upregulate the production of complement component 3, disrupting the blood brain barrier (BBB), causing entry of plasma growth factors into the CSF and promoting cancer growth [7]. Only 5% of patients with breast cancer develop leptomeningeal involvement. However, breast cancer remains the most common etiology of LM [1]. Specific to breast cancer, lobular carcinomas have a higher tendency to metastasize into the meninges when compared to ductal carcinoma, especially triple-negative breast cancer (TNBC). It is estimated that up to 40% of patients with LM have TNBC and TNBC is 3.5 times more common among patients with LM than compared to all individuals with breast cancer [8]. LM presents later in the disease course, with a reported median time from the initial breast cancer diagnosis of 7.4 years [9].

Imaging is crucial for the diagnosis of LM. MRI of the complete neuroaxis, which has an estimated sensitivity of 66-98%, is required to evaluate whether both the brain and spinal cord are involved [6]. Findings commonly seen on MRI are meningeal and/or nerve root enhancement, sulcal enhancement or obliteration, and lesions in the ependymal space and/or vertebral canal [6]. Lumbar puncture is recommended, which often reveals mild pleocytosis with elevated protein and hypoglycorrhachia [3]. The gold standard of LM diagnosis is the presence of malignant cells in the CSF by cytology [2]. CSF cytology is positive in over 90% of patients with suspected LM after three high-volume lumbar punctures, and specificity is over 95% [3]. The prognosis of patients with breast cancer and LM remains poor, even in patients receiving aggressive treatment. Studies show that median overall survival from the time of LM diagnosis ranges from seven to 21 weeks [10].

Guillain-Barre syndrome (GBS), or an acute inflammatory demyelinating polyradiculoneuropathy (AIDP), is commonly preceded by infection (in 76% of patients) and followed by progressive limb weakness, which can last up to four weeks [5]. GBS has historically been known for diagnostic CSF studies with an albuminocytologic dissociation. However, normal CSF studies do not rule out a diagnosis of GBS because 30-50% of patients have normal protein CSF in the first week and 10-30% of patients in the second week [11]. To support this diagnosis, nerve conduction studies are typically done, which can show polyradiculoneuropathy and CSF studies typically show an increase in protein with normal white blood cell count [5]. The management of patients with Guillain-Barre syndrome is supportive care of complications that arise, intravenous immunoglobulin (IVIG), and plasma exchange [5]. IVIG and plasma exchange have been shown to improve disease outcomes by expediting recovery but they do not prevent the progression of disease or improve the extent of nerve damage [5].

## Conclusions

The similar symptomatology of GBS and leptomeningeal carcinomatosis can lead to misdiagnosis and a delay in treatment, which is especially problematic in oncologic patients. Proper diagnosis and treatment allow cancer patients accelerated recovery of GBS and, hopefully, accelerated return to cancer treatment. In our case, based on the history of recent diarrhea, careful physical examination, and MRI findings, GBS was the most likely diagnosis. Prompt initiation of IVIG allowed clinical improvement. Having a high index of suspicion for more reversible conditions, such as GBS, allowed the regain of function in our patient with metastatic breast cancer.
